# Mesenchymal Stromal Cells Implantation in Combination with Platelet Lysate Product Is Safe for Reconstruction of Human Long Bone Nonunion

**DOI:** 10.22074/cellj.2016.4557

**Published:** 2016-08-24

**Authors:** Narges Labibzadeh, Mohsen Emadedin, Roghayeh Fazeli, Fatemeh Mohseni, Seyedeh Esmat Hosseini, Reza Moghadasali, Soura Mardpour, Vajiheh Azimian, Maede Ghorbani Liastani, Ali Mirazimi Bafghi, Mohamadreza Baghaban Eslaminejad, Nasser Aghdami

**Keywords:** Fractures Ununited, Mesenchymal Stromal Cells, Platelet Lysate

## Abstract

**Objective:**

Nonunion is defined as a minimum of 9 months since injury without any visible progressive signs of healing for 3 months. Recent literature has shown that the application of mesenchymal stromal cells is safe, *in vitro* and *in vivo*, for treating long bone nonunion. The present study was performed to investigate the safety of mesenchymal stromal cell (MSC) implantation in combination with platelet lysate (PL) product for treating human long bone nonunion.

**Materials and Methods:**

In this case series clinical trial, orthopedic surgeons visited eighteen patients with long bone nonunion, of whom 7 complied with the eligibility criteria. These patients received mesenchymal stromal cells (20 million cells implanted once into the nonunion site using a fluoroscopic guide) in combination with PL product. For evaluation of the effects of this intervention all the patients were followed up by taking anterior-posterior and lateral X-rays of the affected limb before and 1, 3, 6, and 12 months after the implantation. All side effects (local or systemic, serious or non-serious, related or unrelated) were observed during this time period.

**Results:**

From a safety perspective the MSC implantation in combination with PL was very well tolerated during the 12 months of the trial. Four patients were healed; based on the control Xray evidence, bony union had occurred.

**Conclusion:**

Results from the present study suggest that the implantation of bone marrow-derived MSCs in combination with PL is safe for the treatment of nonunion. A double blind, controlled clinical trial is required to assess the efficacy of this treatment (Registration Number: NCT01206179).

## Introduction

It is estimated that nonunion occurs in approximately 5-10% of fractures (1). Atrophic nonunion remains the most difficult to treat, even with autologous bone grafting, the current treatment of nonunion, and can lead to numerous operations and socioeconomic costs (2). There exist numerous alternative testable treatments for nonunion (3,4) but none of these have been approved so far. In the past few decades, there have been a large number of studies of stromal cell applications in regenerative medicine. These have focused mainly on Mesenchymal stromal cells as non-hematopoietic stromal cells which are present in some human tissues and have multilineage differentiation ability (5,6). This ability is an ideal option for treating bone defects such as nonunion. Several experimental studies and a number of human clinical trials have already indicated the safety and efficacy of mesenchymal stromal cells (MSCs) in the treatment of nonunion (7,11). Xue et al. (12) reported the successful use of intravenously infused umbilical cord mesenchymal stromal cells to treat the gap in the bone and improve nerve conduction velocity in one patient with nonunion of the humerus and radial nerve injury, and Murena et al. (13) treated two cases of aseptic humeral shaft nonunion by using opposite structural allograft, bone morphogenic protein 7 and MSCs. Through the expression of several growth factors from activated thrombocytes, the application of platelet derived products, for instance platelet lysate (PL) product, stimulates regeneration thus stimulating recovery through cell application (14,15). In this method regeneration to osteocytes would occur. 

The present study was conducted to evaluate the safety of MSC implantation in combination with PL, as the source of growth factors for stimulating the MSCs to convert into bone cells (16) for the treatment of human long bone nonunion. 

## Materials and Methods

### Patients

**Fig.1:**
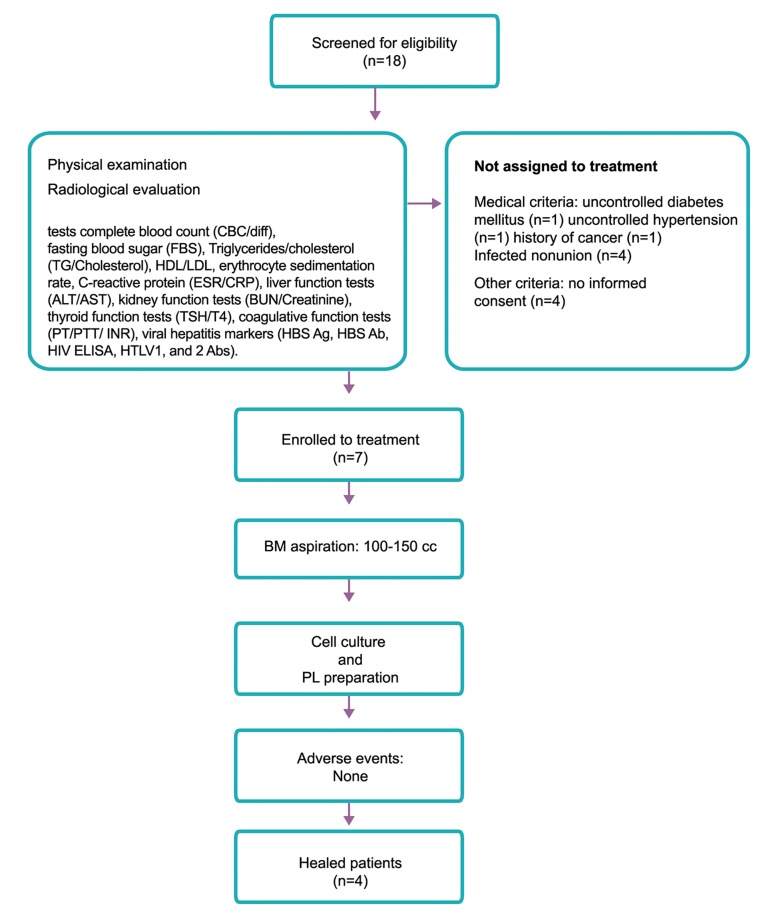
Flow of patients through study. BM; Bone marrow, MSCs; Mesenchymal stromal cells, PL; Platelet lysate, HDL; High-density lipoprotein, LDL; Low-density lipoprotein, ALT; Alanine transaminase, AST; Aspartate aminotransferase, BUN; Blood urea nitrogen, TSH; Thyroid stimulating hormone, T4; Thyroxin, PT; Prothrombin time, PTT; Partial thromboplastin time, INR; International normalized ratio, HBS Ag; The surface antigen of the hepatitis B virus, HBS Ab; Hepatitis B antibody, HIV; Human immunodeficiency virus, and HTLV; Human T-lymphotropic virus or hu- man T-cell lymphotropic virus.

As a case series clinical trial, the study was approved by the Ethical Review Board of Royan Institute. (Ethical Permission number: NCT01206179). Informed consent was taken from all eligible patients before inclusion in the study. Seven patients were selected from eighteen patients by orthopedic surgeons based on inclusion and exclusion criteria between 2012 and 2013 (Fig.1, Table1). 

### Isolation and characterization of bone marrow mesenchymal stromal cells

The operations were performed under sterile conditions in the operating room under local anesthesia with 2% lidocaine solution and sedation by intravenous infusion with midazolam, 0.1 mg per kg and fentanyl 25-50 mg per 100 mm. Bone marrow was aspirated from both iliac crests by a hematologist/ oncologist specialist. Samples were transferred to the clean rooms of Royan Institute less than 2 hours later. Mononuclear bone marrow cells were isolated under sterile conditions according to the density gradient strategy by Ficoll-Paque open system (Lymphodex, Inno-TRAIN, REF: 002041600). The next step was performed by isolating and washing the mononuclear cell layer in phosphate-buffered saline (PBS) buffer (Miltenyi Biotech GmbH, REF: 700-25, 1:1). Cell count and cell viability was evaluated using trypan blue staining and confirmed using the NucleoCounter system (ChemoMetec A/S, Denmark). Mononuclear cells were then cultured under standard culture conditions consisting of MEM Alpha Medium 1X (Gibco, Germany, Cat No: 22571) supplemented with 10% fetal bovine serum (17) Pharma Grade (PAA, Cat No: A15-512), and were then seeded with 1×10^6^ mononuclear cells (MNCs)/cm^2^ in Millicell HY Flasks (Millicell HY Flask T-600, Cat No: PFHYS0616) for primary culture. Flasks were incubated under pre-defined conditions, including 5% CO_2_ at 37˚C. 

Following the initial 3-4 days, the medium was transferred to new flasks in order to give the floating cells enough time to attach. Non-adherent cells were removed by changing the culture medium after 3-4 days, a process that was repeated every 3 days. After one or two passages, fibroblast-like cells were harvested at 90% confluence by applying 0.25% trypsin in 0.1% EDTA. Cell viability was determined by trypan blue staining as well as by the NucleoCounter system before injection. 

**Fig.2:**
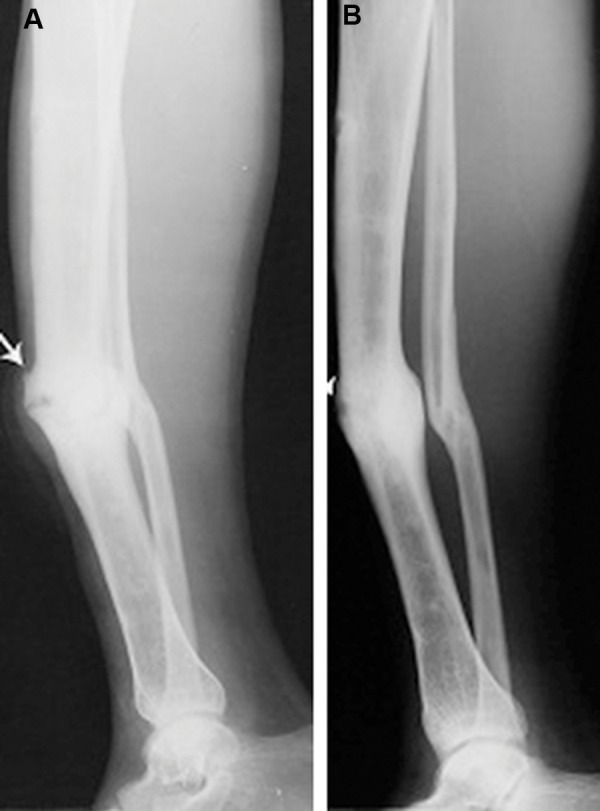
Lateral radiograph of a 32 years male non-united tibial fracture, arrow shows the fracture. A. Before intervention and B. 6 months after using MSCs in combination of platelet lysate product, arrow shows radiological signs of healing. MSCs; Mesenchymal stromal cells.

Flow cytometry analysis was performed in order to determine the expression of cell surface markers. The characterization panel consisted of monoclonal antibodies for mesenchymal lineage markers, including CD90-FITC (EXBIO, Cat No: 1F-652-T100), CD105-PE Endoglin (BD Pharmingen^TM^, Cat No: 560839), CD73-PE (BD Pharmingen^TM^, Code No: 550257), CD44-FITC (BD Pharmingen^TM^, Code No: 555478), and CD45FITC-CD34PE (BD Pharmingen^TM^, Cat No: 341071), and isotype controls, including MultiMixTM FITC Mouse IgG1, PE-Mouse IgG1 (X0932, Dako), FITC-Mouse IgG2b (Millipore, Cat NO: MABC006F), PE conjugated Mouse IgG1k (BD Pharmingen^TM^, Cat NO: 551436). Cells were fixed with 4% paraformaldehyde and immunophenotyping analysis was performed using a BD FACS Calibur flow cytometry system (BD Biosciences, USA). Finally, cells were resuspended in 7 ml normal saline supplemented with 2% human serum albumin (Octalbin, Octapharma, AG, Seidenstrass2 CH-8853 Lachen, Switzerland). Figure 2 indicates the characterization of MSCs. 

**Table 1:** Inclusion and exclusion criteria


Inclusion criteria	Exclusion criteria

18<age<65 Y	Active infection at nonunion site
Established nonunion of femur or tibia	Inadequate fixation of nonunion
Diaphysial	Positive viral tests
Atrophic type nonunion	Pregnancy, lactating Chronic, uncontrolled diseases


#### Platelet lysate preparation

Umbilical cord blood (UCB) was collected from human and centrifuged at 2000 g for 2 minutes or 1000g for 15 minutes at 20˚C. Then the platelet rich plasma (PRP) was collected and centrifuged at 3000 g for 10 minutes at 10˚C. After that it was frozen at -70˚C overnight. Next day the platelets were thawed in a water bath (37˚C) and heat inactivated at 56˚C for 30 minutes. Finally, they were centrifuged at 900 g for 30 minutes and removed PL bodies. Aliquots of 5-10 ml were stored at 70˚C.

#### Preparation of cells for implantation

Primary cultures of MSCs were washed with PBS and trypsinized with trypsin/EDTA (0.05%, Gibco, Germany, Cat No: 25300-062). The cells were suspended in normal saline at a density of 10×10^6^/ml medium and loaded into 10 ml sterile syringes. For each patient, about 20-50×10^6^ cells were prepared and then were taken to the hospital in a cold box at 4˚C. The MSCs suspension was mixed with the PL product in 3 ml volume and then implanted into the injured site under the same conditions.

#### Early Side effects evaluation

Adverse events were divided into local or systemic, serious or non-serious, related or unrelated. Local adverse events were those limited to the nonunion site; systemic ones were those unrelated to the nonunion site; serious adverse events included death, neoplasms, infections, pulmonary embolisms, and anaphylactic shock. These early side effects were evaluated for all patients immediately after the implantation and 3, 6, and 12 months after that. At these intervals X-rays of the affected limb, as well as the above mentioned laboratory tests, were examined.

### Results

**Fig.3:**
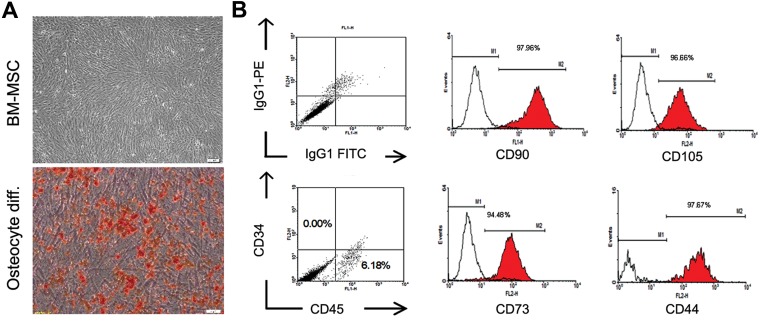
Characterization of passaged 1 human BM-derived MSCs. A. Phenotypic appearance and osteogenic differentiation potential of pas- saged one BM-derived MSCs (Alizarin red staining) and B. Representative flow cytometric analysis using WinMDI software indicated the expression of CD90, CD105, CD73, CD44 and CD45/CD34 surface markers (Red lines) on MSCs of both groups compared to isotype controls (Black lines). BM-MSCs; Bone marrow-mesenchymal stromal cells.

The basic characteristics of the patients are defined in Table 2. Improvement in healing and bone union was seen in four patients. One was a 26-year- old man with closed fracture of the femur, which had caused deformity in the lower limb which had been unsuccessfully treated by plating. Six months after the MSCs plus PL implantation, union was observed in the radiological assay. The second patient was a 32-year-old man with an open fracture of tibia that had led to tenderness at the site and nonunion for 24 months. However, six months after the implantation, union occurred (Fig.3) In the third patient, a 48-year- old man with an open fracture of the fibula that had remained un-united for 8 months, healing occurred two months after the implantation. The last patient was a 46-year-old woman with a closed fracture of the femur. In this case nonunion had lasted for 3 years causing shortening of the lower limb. However, 12 months after the implantation of MSCs plus PL, union occurred as confirmed by X-ray examination. According to hematological, biochemical, serological laboratory tests there were no side effects of MSCs implantation. Three patients did not benefit from this implantation. They had had nonunion for 11 years, 4 years, and 16 months in femur, femur, and both bones of the leg, respectively (Table 2). It is noteworthy that we did not observe any side effects in either group.

#### Discussion

305 MSCs in Combination with PL in Nonunion Discussion Recent advances in cell biology have been amalgamated in the new discipline of bioengineering of different cell co-products. In addition tissue engineering, with different scaffolds have been used in several clinical settings so far (18,20). All of these treatments need surgical procedures and cause extra costs for both physician and patient, so finding a new kind of less invasive treatment would be a promising approach for treating long bone nonunion. However, the translation of this rapidly expanding discipline to clinical practice is limited so far, and this is the first report of treating long bone nonunion using cell biology in combination with PL product. The successful combination of bone marrow derived MSCs (BMSCs) and PL as a cell stimulator has not to our knowledge been reported in the clinical setting. Stromal cell therapy is a new testable approach for treating long bone nonunion (18,22). Mesenchymal stromal cells, as the pluiripotent progenitor cells, which can differentiate into various cell types, including osteoblasts, and are easy to expand *in vitro* culture, are ideal for this purpose (23,24). There is some experimental and clinical evidence to support the safety and efficacy of BMSCs in enhancing osteogenesis (20,25,26). 

However, osteoprogenitor cells may be insufficient in quantity or unable to recognize cellular cues at the site of nonunion, especially atrophic sites. 

Thus, local implantation with a suitable number of active and viable cells can work, not only by local osteogenesis but also by stimulation of osteoblastic differentiation of native cells by releasing signaling molecules. In this way endogenous fracture healing mechanisms are activated (19). 

**Table 2:** Demographic and clinical characteristics of the patients included in the study


Case	Age	Sex	Site	Initial injury	Duration of nonunion	Physical exam	Fracture mobility	Initial treatment	Type of nonunion	Time of union *

1	26	M	Femur	Closed	24 m	Deformity	Yes	Plating	A	6 m
2	32	M	Tibia	Open	24 m	Tenderness	No	Plating	A	6 m
3	48	M	Fibula	Open	8 m	Deformity	Yes	Plating/ext. fixation	A	2 m
4	48	F	Femur	Open	11 Y	Deformity	No	Plating	A	Failed to unite
5	52	M	Femur	Closed	4 Y	Deformity	No	Plating	A	Failed to unite
6	46	M	Femur	Closed	3 Y	Shortening	No	Int. fixation	A	12 m
7	61	F	Tibia and fibula	Open	16 m	Shortening	No	Plating/int. fixation	A	Failed to unite


*; Time of union is defined based on the radiological (anteroposterior and lateral views of radiography evaluated by an independent radiologist and defined as bridging callus formation and absence of fracture line at the site of more than three out of four cortices, in undiagnosed cases computed tomography was used) and clinical results (no tenderness and pain at the fracture site with weight bearing) at the mentioned time frames, m; Month, and Y; Year.

### Discussion

Recent advances in cell biology have been amalgamated in the new discipline of bioengineering of different cell co-products. In addition tissue engineering, with different scaffolds have been used in several clinical settings so far (18-20). All of these treatments need surgical procedures and cause extra costs for both physician and patient, so finding a new kind of less invasive treatment would be a promising approach for treating long bone nonunion. However, the translation of this rapidly expanding discipline to clinical practice is limited so far, and this is the first report of treating long bone nonunion using cell biology in combination with PL product. The successful combination of bone marrow derived MSCs (BMSCs) and PL as a cell stimulator has not to our knowledge been reported in the clinical setting. Stromal cell therapy is a new testable approach for treating long bone nonunion (18-22). Mesenchymal stromal cells, as the pluiripotent progenitor cells, which can differentiate into various cell types, including osteoblasts, and are easy to expand *in vitro* culture, are ideal for this purpose (23, 24). There is some experimental and clinical evidence to support the safety and efficacy of BMSCs in enhancing osteogenesis (20, 25, 26). However, osteoprogenitor cells may be insufficient in quantity or unable to recognize cellular cues at the site of nonunion, especially atrophic sites. Thus, local implantation with a suitable number of active and viable cells can work, not only by local osteogenesis but also by stimulation of osteoblastic differentiation of native cells by releasing signaling molecules. In this way endogenous fracture healing mechanisms are activated (19).

According to several studies bone marrow aspiration can promote bone healing in cases of nonunion; the quantity of progenitor cells being an important parameter in such cases (27,29). However, the quantity of cells in bone marrow aspirate is less than 0.01% and cell concentration and culture would be a promising approach for improving results (30). It has been discussed previously that cell culture could cause loss of the “supporting cells” in the bone marrow aspirate, but it also has advantage of increasing the quantity of progenitor cells which specifically turn into host cells at the site of injury, including nonunion sites (19). Quarto et al. (20) showed in a clinical setting that osteoprogenitor cells from bone marrow grown on ceramic scaffold, with external fixation initially for mechanical stability, could be used to cure 3 long term cases of nonunion, which had not responded to previous routine treatments. Marcacci et al. (31) reported complete fusion of four cases with large bone diaphysis defects, 5-7 months after implantation of autologous bone marrow stromal cells expanded in culture and seeded onto porous hydroxyapatite (HA) ceramic scaffolds. They followed their patients for 6-7 years after stromal cell therapy and there were no adverse events. Although these initial studies suggest the potential usefulness of stromal cell therapy in nonunion, additional clinical trials are necessary to evaluate safety and efficacy of this treatment. 

Platelet derived products, which provide a reservoir of different growth factors and cytokines, are thus suitable for stimulating the expansion of resting osteoblasts and so can be considered as a good therapeutic product in regenerative medicine for bone repair (32). As Kitoh et al. (33) reported, using culture-expanded bone marrow cells (BMCs) and platelet-rich plasma (PRP) during limb lengthening shortens the treatment period by accelerating callus formation. 

Results from the present study revealed that the harvest, isolation and implantation of MSCs in combination with PL is safe for treating bone nonunion, and, in some cases, can improve the healing process. There was no identification of malignant tumors or any other complications during the study. These outcomes indicate the feasibility and overall safety of stromal cell therapy in combination with PL in patients with nonunion. The present work was a prospective clinical trial with a small number of cases, therefore, we could not perform any statistical analysis for evaluating its efficacy for healing bone nonunion. Similarly, because of the small size of the trial, evaluation of the patients not responding to treatment was also impossible. One hypothesis is that long duration of nonunion and older age of the patient might adversely influence the healing process (34,35). However, because of the problematic treatment of nonunion, it seems that MSCs in combination with PL product are useful for treating this condition. Future randomized studies with larger sample sizes are required to achieve statistical significance. 

### Conclusion

Bone nonunion is a morbid disease that involves both patients and physicians, and, consequently, affects society. Results from the present study suggested, for the first time, that the harvest, isolation, and transplantation of autologous bone marrow derived MSCs in combination with PL product is feasible and safe overall for treating bone nonunion. Cases treated successfully in the current study include some that had not responded to the iliac bone grafting method. Use of MSCs is a rapidly expanding focus of research in all fields of medical science, in particular in the field of orthopedics, and needs further scientific investigation. Future randomized clinical trials with larger sample sizes are necessary to evaluate the efficacy of MSCs in combination with PL implantation for the treatment of long bone nonunion. 
